# Abortion Storms in Dromedary Camels: Causes, Pathogenesis, Risks, Diagnosis, and Control

**DOI:** 10.3390/ani16213383

**Published:** 2026-10-27

**Authors:** Ahmed Ali, Derar R. Derar

**Affiliations:** Department of Clinical Sciences, College of Veterinary Medicine, Qassim University, Buraydah 51452, Saudi Arabia; dr.mohammad@qu.edu.sa

**Keywords:** dromedary camel, abortion storms, epidemiology, infectious, reproduction

## Abstract

Abortion storms are a significant reproductive and economic challenge for dromedary camels, especially in the Arabian Peninsula and North Africa. They are characterized by an abrupt increase in pregnancy losses within a short period of time. The camel’s seasonal breeding pattern, which restricts opportunities for rebreeding within the same reproductive season, amplifies their impact. Abortion storms are multifactorial phenomena that arise from intricate relationships between infectious agents, host susceptibility, environmental factors, and management techniques. Bacterial pathogens like *Brucella* spp., *Chlamydia abortus*, and *Coxiella burnetii*, as well as protozoal infections like *Trypanosoma evansi* and *Toxoplasma gondii* are thought to be the main infectious causes. However, viral, fungal, nutritional, environmental, and management-related factors may also play a role. This narrative review addresses the biological mechanisms underlying herd-level outbreaks and summarizes the current understanding of the etiology, pathogenesis, and risk factors related to abortion storms in dromedary camels. Relevant literature was identified through structured searches of PubMed, Scopus, and Web of Science, supplemented by Google Scholar and reference-list screening, covering published evidence on abortion and abortion storms in dromedary camels. It offers helpful advice for field research, clinical assessment, sample collection, laboratory diagnosis, outbreak response, and long-term prevention in addition to addressing current diagnostic issues. In order to lessen the frequency and effects of abortion storms in camel populations, standardized diagnostic procedures, enhanced surveillance systems, and integrated herd health strategies are highlighted as key knowledge gaps and future research priorities.

## Simple Summary

Abortion storms in dromedary camels are sudden increases in pregnancy losses that cause major economic problems, especially in the Arabian Peninsula and North Africa. Because camels breed only during certain seasons, lost pregnancies cannot be replaced quickly. These storms are caused by many factors working together, including bacteria such as *Brucella*, *Chlamydia*, and *Coxiella*, parasites such as *Trypanosoma* and *Toxoplasma*, plus environmental stress, poor nutrition, and management problems. This narrative review explains what causes these outbreaks, how they develop, and what factors increase the risk. It provides practical advice for veterinarians and camel owners on how to investigate outbreaks, collect samples for laboratory testing, and prevent future losses. Early action and good herd management are the keys to reducing the impact of abortion storms. The review also identifies gaps in current knowledge and suggests future research priorities to improve diagnosis and control.

## 1. Introduction

In recent years, recurrent episodes of clustered abortion have been reported in camel-rearing regions, particularly in the Arabian Gulf, suggesting that abortion storms may represent an important reproductive problem [1,2,3]. These storms have significant economic ramifications because dromedary camels are seasonal breeders with a short breeding season [4,5,6,7].

Infectious and non-infectious factors interact to cause camel abortion, a multifactorial condition. Because they can spread quickly within herds, infectious agents, such as bacterial pathogens (like *Brucella* spp., *Chlamydia abortus*, and *Coxiella burnetii*), protozoa (like *Trypanosoma evansi*, *Toxoplasma gondii*, and *Neospora caninum*), and, less frequently, viral infections, are frequently associated with abortion in dromedary camels and may contribute to abortion storms [8,9,10,11,12,13,14,15,16]. Heat stress, nutritional deficiencies, mycotoxins, and endocrine disorders are examples of non-infectious factors that may either directly cause or increase the risk of infectious abortion [17,18,19].

The epidemiology, pathophysiology, and risk factors of abortion storms in camels are still poorly understood, despite an increase in reports of abortion outbreaks. The majority of studies that are currently available are regional, have small case numbers, and employ a variety of diagnostic techniques, which makes comparisons challenging and probably contributes to underreporting.

Unlike previous reviews that have primarily catalogued infectious causes of abortion in camelids, this review integrates (i) the causes of abortion, (ii) the herd-level epidemiological and biological cascade that converts endemic infection or individual risk factors into abortion storms, (iii) the diagnostic and interpretive challenges that complicate etiological attribution, and (iv) practical, field-oriented outbreak investigation and control measures. It also explicitly distinguishes camel-specific evidence from evidence extrapolated from other livestock species and separates exposure, association, direct pathogen detection, and etiological involvement.

## 2. Literature Search Approach

A structured literature search was conducted to identify published evidence relevant to abortion and abortion storms in dromedary camels. The search focused primarily on PubMed, Scopus, and Web of Science, with additional searches of Google Scholar when required to identify relevant publications not retrieved from the primary databases. The main search terms included combinations of “dromedary camel,” “camel,” “abortion,” “abortion storm,” “pregnancy loss,” “reproductive failure,” “stillbirth,” “infectious abortion,” “Brucella,” “Chlamydia,” “Coxiella,” “Trypanosoma,” “Toxoplasma,” “Neospora,” “viral infection,” “diagnosis,” “epidemiology,” “treatment,” “control,” and “prevention.” The search covered the available literature from the earliest relevant reports through 2026, with particular emphasis on recent studies and current evidence. Studies were considered relevant when they provided information on the occurrence, epidemiology, potential causes, diagnostic evidence, risk factors, management, or prevention of abortion in dromedary camels. Reference lists of relevant articles and review papers were also examined to identify additional studies. As this manuscript is a narrative review, the search was intended to provide broad and current coverage of the available evidence rather than to follow a formal systematic-review protocol or quantitative meta-analysis.

## 3. Definition of an Abortion Storm

In this review, an abortion storm is defined operationally as an unusual temporal or spatial clustering of abortions within a camel herd, representing an increase above the herd’s expected background occurrence over a relatively short period and suggesting a common infectious, environmental, or management-related exposure. Because no universally accepted numerical threshold has been established specifically for dromedary camels, the occurrence of a storm should be interpreted in relation to herd size, number of pregnant females at risk, historical abortion frequency, gestational stage, and the temporal distribution of cases. Accordingly, the literature summarized in Table 1 includes both documented herd-level abortion outbreaks and studies reporting individual abortions or evidence of exposure to potential abortifacient agents; these levels of evidence should not be considered equivalent.

## 4. Evidence Strength and Interpretation

Evidence linking a pathogen to abortion in dromedary camels has been interpreted according to the type and strength of available evidence. Serological positivity primarily indicates exposure and, by itself, does not establish that the detected agent caused abortion. Stronger evidence is obtained when the pathogen is directly detected in fetal, placental, or reproductive tissues, particularly when molecular or culture-based detection is accompanied by compatible gross or histopathological lesions and an appropriate epidemiological pattern. Evidence of association may be strengthened by repeated detection among affected animals, temporal or spatial clustering, and biological plausibility; however, pathogen detection alone does not necessarily demonstrate causation. Accordingly, this review distinguishes between evidence of exposure, epidemiological association, direct pathogen detection, compatible pathological findings, and stronger evidence supporting etiological involvement. This distinction is particularly important in abortion storms, where co-infections and non-infectious factors may contribute to pregnancy loss.

## 5. Causes of Abortion

Abortion storms are most often associated with infectious agents capable of transmission within herds, although environmental, nutritional, and management factors may predispose to or amplify outbreak occurrence.

### 5.1. Infectious Causes

Abortion storms in dromedary camels are predominantly associated with infectious agents (Table 1), with bacterial pathogens being the most frequently documented, followed by viral and protozoal agents. Environmental and management stressors may facilitate disease expression and transmission, while co-infections can further complicate the clinical outcome.

#### 5.1.1. Bacterial Causes

Bacterial infections are among the most frequently reported and investigated infectious causes of abortion in camels, particularly in endemic regions such as the Middle East and North Africa. Among these, *Brucella* spp. (primarily *Brucella melitensis* and *Brucella abortus*) are among the more frequently reported causes of abortion in dromedary camels. The disease is globally distributed and has major economic and zoonotic implications [11,15,28,29,30,31,32,33,34]. Infected females typically exhibit late-term abortion, retained placenta, and reduced fertility [35,36]. The pathogenesis of brucellosis is closely linked to the affinity of the bacteria for reproductive tissues, particularly the placenta, where erythritol-rich environments favor bacterial proliferation, leading to placentitis and fetal death [34,35,37,38]. Recent studies confirm that camel brucellosis remains endemic in many camel-rearing regions, with seroprevalence varying widely and reaching significant levels in some populations, emphasizing its role in herd-level reproductive losses and potential abortion outbreaks [30,32,33,34]. According to the available literature, the seroprevalence of *Brucella* infection in dromedary camels shows considerable variation, ranging from 2.9% to 22.8% across different regions [11,15,28,32,39]. This variability is largely influenced by geographic location, endemicity, seasonality, stress conditions, history of abortion, general health status, management system (nomadic vs. intensive), interspecies contact, farm biosecurity, age, and nutritional status. A Jordanian study specifically investigated *Brucella*-associated abortion in dromedary camels and reported a 1.9% incidence of Brucella-caused abortion, with *B. melitensis* isolated from four aborted fetuses [38]. A recent review by Abdel-Rady et al. [32] reported an even wider global range of 0.4% to 37.5%. Notably, seroprevalence appears to follow two distinct epidemiological patterns: lower rates (2–5%) in nomadic or extensively managed camels and higher rates (8–15%) in intensively or semi-intensively managed systems [9]. Intensive and semi-intensive systems may facilitate transmission when animals are kept in closer proximity and when multiple animals are repeatedly exposed to contaminated reproductive materials or environments. In contrast, nomadic or extensively managed camels may experience lower sustained animal density and less prolonged contact within a fixed herd. Regionally, countries such as Sudan and Saudi Arabia are among the most affected, reflecting the endemic nature of brucellosis in these areas [32]. Despite its recognized importance, there is still no universally standardized, camel-specific policy for the control of brucellosis across camel-producing countries. Control measures are often fragmented or adapted from other livestock systems, leading to inconsistent implementation and effectiveness [9,32,39].

Chlamydiosis is recognized as a potential cause of abortion in *Camelus dromedarius* across the Middle East and Africa [2,10,39]. Reported seroprevalence rates in Saudi Arabia range from 10 to 20%, influenced by herd structure and diagnostic methods [13,40], while higher seropositivity (22.6%) has been observed in aborting, particularly older, females in Tunisia [15]. These cases may have involved other infectious agents, co-infections, or non-infectious factors. A well-documented abortion storm in Saudi Arabia showed that 33.8% of pregnant females aborted within a short period, with *Chlamydia abortus* confirmed by PCR in both females and breeding males, suggesting possible reproductive tract involvement and raising the possibility of male-to-female transmission [2]. Similar findings across North Africa and the Arabian Peninsula indicate widespread circulation of this pathogen [2,15,41]. In addition to abortion, associated conditions such as retained placenta, metritis, and infertility contribute significantly to long-term reproductive losses, and severe cases may progress to systemic illness [39,40,41].

*Coxiella burnetii*, the causative agent of Q fever, has been associated with abortion in dromedary camels, with notably high seroprevalence reported across Africa and the Arabian Peninsula. Seropositivity ranges widely from 22% to over 60% in countries such as Egypt, Saudi Arabia, and Sudan, and may reach 70–80% in regions like Algeria and Chad, often exceeding rates observed in other ruminants [1,13,42]. Infection is consistently associated with key risk factors, including advanced age, previous abortion, contact with small ruminants, and heavy tick infestation, particularly involving *Hyalomma* spp. Seasonal increases, especially during autumn and winter, have also been reported [42]. Molecular evidence further supports its etiological role, with *C. burnetii* detected in 36% of uterine samples from aborting camels, often alongside other pathogens, highlighting the potential for co-infections [13]. In addition to its reproductive impact, the pathogen’s persistence in camel tissues and its zoonotic nature underscore its significance for both herd health and public health [1].

Other bacterial pathogens reported as causes of abortion in camels include *Leptospira* spp., *Listeria monocytogenes*, *Bacillus cereus*, *Campylobacter* spp., *Salmonella* spp., *Escherichia coli*, and *Streptococcus equi* subsp. *zooepidemicus* [13,19].

#### 5.1.2. Protozoal Causes

*Trypanosoma evansi*, the causative agent of surra, is a major protozoal pathogen implicated in reproductive failure in dromedary camels, particularly in tropical and subtropical regions. Beyond its well-known systemic effects, accumulating evidence supports its role as a potential cause of abortion, especially during epizootic outbreaks. Molecular detection studies have identified *T. evansi* DNA in up to 16.8% of aborted fetuses, confirming its direct association with fetal loss [8,12,43,44,45]. In severe herd outbreaks, abortion rates may reach exceptionally high levels, with reports of up to 80–94% pregnancy loss in affected animals [1,43,44]. The pathogenic mechanisms are multifactorial, involving anemia, immunosuppression, and systemic stress, which compromise pregnancy maintenance, as well as possible endocrine disruption and placental pathology. Transmission occurs primarily via mechanical vectors such as biting flies, but vertical transmission may also contribute to reproductive losses. Overall, *T. evansi* should be considered a key etiological agent in camel abortion storms, particularly under conditions favoring vector abundance and herd susceptibility.

*Toxoplasma gondii* (*T. gondii*) is a widespread protozoan parasite with recognized abortifacient potential across multiple species, and growing evidence suggests its involvement in reproductive losses in camels. Seroprevalence studies indicate moderate exposure levels, ranging from 15% in Iran, 25.7% in Egypt, and 49.14% in Saudi Arabia [14,46,47]. Field investigations in Algeria reported a high herd-level occurrence of abortion (80%) and stillbirth (60%), with a significant association with the presence of domestic cats, which are the definitive hosts of *T. gondii* and the principal source of environmental oocyst contamination [25]. Histopathological findings, including detection of tissue cysts and tachyzoites, support a potential etiological role, although direct evidence remains limited. Overall, *T. gondii* is likely an underrecognized contributor to sporadic abortion in camels, particularly in environments with high exposure to definitive hosts.

Other protozoal parasites have been reported in camels, but their role in abortion has not been clearly established. *Neospora caninum*, a well-known cause of abortion in other camelids such as alpacas and llamas, has not been consistently detected in dromedary tissues, suggesting a limited or uncertain role in this species [16,19,25,48]. *Tritrichomonas fetus* has been isolated from dromedaries; however, there is no strong evidence linking it to abortion [49]. Similarly, although sarcocystosis is widespread, its contribution to reproductive loss in camels has not been clearly established [50].

#### 5.1.3. Viral Causes

Viral infections are less frequently reported but may contribute to abortion in dromedary camels, often under specific epidemiological conditions. During an abortion outbreak in Dubai, 8 of 80 pregnant dromedaries (10%) aborted, and equine rhinitis A virus (ERAV) was isolated from aborted fetuses. Experimental infection of two pregnant dromedaries subsequently resulted in abortion in both animals (2/2; 100%), with the same virus reisolated from the fetuses, providing strong evidence for the abortifacient potential of ERAV [20].

Bovine viral diarrhea virus (BVDV) has been detected serologically in dromedary camels and has been associated with a history of abortion. For example, BVDV seropositivity was 4.8% overall in an Egyptian survey and increased to 13.8% among animals with a previous abortion history [27]. However, these findings indicate exposure and epidemiological association rather than a confirmed BVDV-attributable abortion rate.

Rift Valley fever virus (RVFV), a mosquito-borne zoonotic pathogen, has also been associated with abortion, with seroprevalence reaching 21.5% and risk factors including age, season, and vector exposure [51].

Other viruses, including Newcastle disease virus (NDV), bluetongue virus, camelpox virus, and equine herpesvirus-1 or equine arteritis virus, have been sporadically reported or isolated in association with abortion [19,52]. NDV has also been isolated from an aborted dromedary fetus in Dubai during a concurrent NDV outbreak in a nearby pigeon farm. However, only an individual aborted fetus was investigated, and the number of pregnant animals at risk and total number of abortions were not reported; therefore, a pathogen-specific abortion rate cannot be calculated and a causal relationship remains unconfirmed [52].

#### 5.1.4. Fungal Causes

Mycotic abortion is rarely reported in dromedary camels but may occur under predisposing conditions such as mold-contaminated feed, poor storage practices, and immunosuppression [19,53,54,55,56]. Fungal-related reproductive losses should be distinguished into two biologically distinct conditions: mycotic abortion caused by invasive fungal infection, and reproductive failure associated with ingestion of mycotoxin-contaminated feed. These entities differ fundamentally in pathogenesis, diagnostic criteria, and epidemiological implications.

Mycotic abortion results from hematogenous dissemination of viable fungi, followed by invasion of the placenta and development of fungal placentitis, which compromises placental function and may cause fetal infection or death. In dromedary camels, *Aspergillus* spp. have been detected in aborted fetuses; a real-time PCR survey of 161 camel fetuses in Iran identified *Aspergillus* DNA in 11.8% (19/161) of cases, with *A. fumigatus* being the most commonly implicated species [55]. However, detection of fungal DNA in aborted fetal tissues should be interpreted cautiously, as post-abortion contamination may yield false-positive results, and detection alone does not establish causation without compatible placental or fetal lesions [55]. Diagnosis of mycotic abortion therefore requires histopathological evidence of fungal hyphae within placental or fetal tissues, ideally supported by culture or molecular identification [54].

Mycotoxin-associated reproductive disorders, by contrast, result from ingestion of feed contaminated with biologically active fungal metabolites and do not involve fungal invasion of reproductive tissues. Ochratoxin A contamination of camel feed has been associated with abortion in a Saudi Arabian camel herd, where 8 abortions occurred among 150 camels following detection of ochratoxin A at six times the permissible limit (30 ppb vs. 5 ppb) [57].

### 5.2. Non-Infectious Causes

Non-infectious factors may contribute to abortion and pregnancy loss in dromedary camels, including fetal and placental abnormalities, twinning, umbilical cord abnormalities, uterine or placental dysfunction, endocrine disturbances, nutritional deficiencies, toxic exposures, heat and other environmental stressors, trauma, and maternal disease. However, unlike the overall incidence of abortion, which has been quantified in some intensively managed camel populations, the frequency and relative contribution of individual non-infectious causes remain poorly defined. In a longitudinal study of 4533 pregnancies beyond 60 days of gestation, 229 abortions were recorded, corresponding to an overall abortion rate of 5.05%; however, individual abortions were not systematically attributed to specific non-infectious causes [58].

Camels are adapted to arid environments; however, extreme heat stress, especially when combined with poor management, can impair reproductive performance. Heat stress can disrupt hormonal balance, reduce uterine blood flow, and increase embryonic or fetal loss, particularly in early pregnancy [59,60].

Nutritional deficiencies, particularly those in energy, protein, and essential trace minerals (such as selenium and zinc), are well known causes of reproductive failure in livestock. These deficiencies reduce immune function, disrupt endocrine regulation, and jeopardize fetal development, making animals vulnerable to both infectious and non-infectious abortions. Selenium deficiency has been linked to embryonic death and abortion, whereas zinc deficiency has been linked to poor pregnancy maintenance and fetal viability [61].

Hormonal disturbances, particularly progesterone deficiency, are important predictors of pregnancy failure in camels, as proper luteal function is required to maintain gestation [62,63]. However, reduced progesterone concentrations should be interpreted cautiously, as they may represent a cause, a consequence, or a correlate of pregnancy failure depending on the underlying condition. Camel-specific evidence directly establishing progesterone deficiency as an independent cause of abortion remains limited. Furthermore, twin pregnancies, while uncommon in dromedaries, are frequently associated with increased embryonic or fetal loss due to limited uterine capacity and compromised fetal viability [58,64].

Management and environmental conditions may influence reproductive health and pregnancy outcomes in dromedary camels. Intensive production systems require appropriate herd health management, biosecurity, nutrition, housing, and reproductive management, while inadequate management may increase exposure to infectious and non-infectious health hazards [65]. Pregnancy loss has also been documented under intensive management, although the specific contribution of individual management factors to abortion remains insufficiently characterized [65].

Non-infectious causes of abortion in dromedary camels include toxic plant ingestion, inappropriate use of abortifacient drugs, trauma, and improper obstetrical manipulations. These factors disrupt pregnancy maintenance through mechanisms such as endocrine disruption, placental insufficiency, or direct fetal harm. Similar mechanisms are well documented in other livestock species [66,67], and camelid-specific reviews confirm that these same categories apply to camels [19].

## 6. Pathogenesis of Abortion Storms

The development of an abortion storm can be viewed as a cascade involving pathogen introduction, infection of susceptible animals, facilitating factors, environmental contamination, and amplification of within-herd transmission. Following pathogen introduction, infection may lead to placental or fetal involvement and pregnancy loss. Environmental, nutritional, metabolic, and management stressors may increase susceptibility or facilitate transmission. Abortion or parturition by infected animals can contaminate the environment with infectious materials, increasing exposure of susceptible animals and promoting further transmission. This cycle may result in the temporal or spatial clustering of abortions characteristic of an abortion storm.

The transition from sporadic abortion to herd-level outbreaks reflects a series of biological and epidemiological processes that disrupt pregnancy maintenance across multiple animals in a short period of time [2,3,68]. This process can be thought of as a series of steps that include pathogen introduction, silent transmission within the herd, triggering stressors, rapid escalation of abortion cases, and environmental contamination, followed by feedback amplification.

The first step is usually the introduction of an infectious agent through direct contact with infected animals, contaminated feed or water, or vectors like biting flies in the case of *Trypanosoma evansi* [8,44]. In herds with inadequate biosecurity, highly transmissible pathogens can spread subclinically before clinical symptoms appear.

Affected dams sometimes develop systemic responses, including fever, inflammatory cytokine release, and hematological disturbances [39,68]. These responses can disrupt uterine homeostasis and reduce uteroplacental blood flow, endangering fetal viability [17,69]. Pathogen localization within the placenta is an important step in disease progression. Many abortifacients exhibit placental tropism, resulting in placentitis, edema, necrosis, and impaired maternal-fetal exchange. In infections like brucellosis and Q fever, extensive replication within placental tissues causes severe inflammatory damage and fetal death [1,70].

Fetal infection can occur through hematogenous dissemination or ascending routes, with outcomes varying according to gestational stage, pathogen virulence, and maternal immune status. Fetal death can be caused by hypoxia, direct tissue invasion, or toxin-mediated injury [17,71]. Endocrine disruption also plays an important role, as pregnancy in camels is dependent on adequate progesterone levels [62]. Systemic infection and stress can cause luteolysis, reduced progesterone secretion, and premature uterine contractions, which can lead to abortion [63]. In pregnant camels, experimental endotoxin administration induced abortion through PGF2α-mediated luteal regression and decreased progesterone concentrations [17].

Abortion storms are distinguished by their rapid amplification within the herd, which is fueled by a variety of reinforcement mechanisms. First, aborted fetuses, placental tissues, and uterine discharges contain high pathogen loads, resulting in widespread environmental contamination of soil, feed, water, and housing areas, allowing for rapid transmission [1,13,72]. Second, pregnant animals are more vulnerable due to physiological immunomodulation, metabolic demands, and hormonal changes during gestation, resulting in synchronized vulnerability throughout the herd [63,73]. Third, camels’ seasonal breeding pattern causes a high proportion of animals to be pregnant at the same time and at similar gestational stages, amplifying the impact of pathogen introduction [7]. Finally, environmental and managerial stressors, such as heat stress, nutritional deficiencies, and poor husbandry practices, can suppress immune function in dromedary camels, potentially enhancing pathogen shedding and acting as triggers that convert endemic infections into explosive outbreak scenarios [60,74].

## 7. Risk Factors Associated with Abortion Storms

Animal-related factors may influence abortion risk, with susceptibility varying according to the causative agent (Table 2). In general, older animals tend to be more susceptible than younger ones, likely due to cumulative exposure and increased likelihood of prior infection [15,20,42,43]. The stage of gestation is critical, as early losses are often unnoticed, whereas late gestation is more frequently associated with visible abortion storms, particularly for pathogens with placental tropism [34]. Immune status is also pivotal; immunocompromised or stressed animals are more likely to develop infection and placental pathology, while pregnancy-related immunomodulation increases herd-level vulnerability [73,74].

Herd-level factors strongly shape outbreak dynamics. Large herd size and high stocking density promote transmission and environmental contamination, while the introduction of new animals without quarantine is a major trigger of outbreaks due to the introduction of asymptomatic carriers or novel pathogens [65,75]. Mixed-species farming further increases risk by facilitating interspecies transmission of key abortifacient agents [15,25,49].

Environmental and seasonal influences are also important. Seasonal breeding causes synchronized pregnancies, which magnifies the impact of pathogen exposure and results in clustered abortion events [7]. Heat stress can impair endocrine function and uteroplacental perfusion, resulting in pregnancy loss, whereas increased vector activity promotes disease transmission, such as trypanosomiasis [60,63,74].

Geographical location and endemicity are important factors influencing abortion risk in dromedary camels, as herds in endemic areas are constantly exposed to abortifacient pathogens through environmental contamination, reservoir hosts, and vector activity. Diseases such as brucellosis, Q fever, and chlamydiosis are common in these areas, resulting in recurring or seasonal abortion outbreaks [1,15]. Climatic conditions and animal movement also play a role in transmission, especially in arid environments where vectors like ticks and biting flies are common [60,74]. As a result, endemicity raises infection pressure and lowers the threshold for abortion storm occurrence.

Biosecurity practices are important determinants. Poor biosecurity, including insufficient isolation and improper disposal of aborted materials, promotes rapid pathogen spread [22,26,33]. Nutritional deficiencies, as well as stressors like transportation or abrupt management changes, weaken immunity and increase the risk of abortion [76,77]. In dromedary camels, transport stress has been shown to elevate cortisol and reduce lymphocyte counts, while nutritional deficiencies in trace minerals and vitamins are associated with immunosuppression and reproductive failure [76,77].

Overall, environmental, seasonal, and geographical factors may modify the risk and magnitude of abortion storms through their effects on pathogen exposure, host susceptibility, and within-herd transmission. Seasonal changes in climate, vector activity, animal movement, feed and water availability, and management practices may interact to create conditions favorable for pathogen transmission and reproductive stress. Similarly, geographical endemicity may reflect persistent pathogen circulation, environmental contamination, animal movement, and local management conditions rather than an independent cause of abortion. These interacting factors may therefore amplify pathogen transmission and abortion occurrence, although their specific contributions and underlying mechanisms remain insufficiently quantified in dromedary camels.

## 8. Diagnostic Challenges in Abortion Storms

Despite advances in diagnostic techniques, establishing a definitive cause of abortion in dromedary camels remains challenging, particularly when aborted fetuses or placental tissues are unavailable, autolysed, or improperly handled. Although low diagnostic success rates have been reported in other camelids such as llamas and alpacas [78], these findings should not be directly extrapolated to dromedary camels. Species-specific data on the proportion of dromedary camel abortions for which a definitive etiological diagnosis can be established remain limited. Low diagnostic success rates are frequently caused by difficulties in detecting, interpreting, and integrating multifactorial evidence in field settings [2,72].

The lack of validated camel-specific diagnostic tools is a major limitation, as most assays, particularly serological tests, are adapted from other livestock species, reducing diagnostic accuracy for diseases such as brucellosis, Q fever, and toxoplasmosis [1,14,15,32,43]. Inadequate sample collection further complicates diagnosis because placental tissues and fetal organs are frequently unavailable, degraded, or handled improperly in the field [2,71,79].

The subclinical and multifactorial nature of infections complicates matters, as seropositivity does not always indicate active infection, and co-infection is common during outbreaks [13]. Although serology is widely used, it cannot reliably distinguish between previous exposure and active disease and may be influenced by cross-reactivity. Molecular methods, such as PCR, provide greater sensitivity but are limited by cost, infrastructure, lack of standardization, and sample quality issues [2,11,12,13,15].

These technical challenges are exacerbated by inadequate surveillance systems and underreporting, limiting the ability to detect and characterize abortion storms [1,3,8]. During outbreaks, rapid case accumulation and environmental contamination frequently result in empirical management without a definitive diagnosis. Finally, establishing causality remains difficult, as detecting a pathogen does not always confirm its role in abortion due to the presence of co-infections and non-infectious factors [13,15,43].

Table 3 compares commonly used diagnostic methods for abortion in camels, highlighting their benefits, limitations, and practical applicability in field settings.

## 9. Control of Abortion Storms

When an abortion storm is suspected, a systematic field investigation should be initiated to determine the extent of the outbreak and identify potential risk factors. The assessment should include the number of pregnant females and abortions, gestational stage, outbreak duration, and distribution of cases, together with herd history, including parity, vaccination status, previous abortions, recent animal introductions or movements, and contact with other livestock. Management and environmental factors, such as nutrition, feed and water quality, hygiene, housing, stocking density, climatic stress, and vector exposure, should also be evaluated. This information provides the epidemiological basis for subsequent clinical examination, sample collection, laboratory diagnosis, and implementation of appropriate control measures [13,25,58].

A comprehensive clinical examination of the aborting dam, aborted fetus, and placenta is essential to identify potential causes and guide laboratory investigations. Examination of the dam should include assessment of general health status, rectal temperature, body condition, and signs of systemic illness, together with reproductive tract evaluation for vaginal discharge, retained fetal membranes, metritis, and other abnormalities. Gross examination of the fetus should document gestational age, degree of autolysis, congenital abnormalities, edema or hemorrhage, and major lesions in organs such as the lungs, liver, spleen, kidneys, heart, and brain. The placenta should be carefully inspected for evidence of placentitis, including cotyledonary necrosis, hemorrhage, exudate, thickening of intercotyledonary membranes, and other gross pathological changes. These findings provide valuable clues for differential diagnosis and help prioritize appropriate laboratory testing [19,71,72].

Appropriate sample collection is critical for establishing a definitive diagnosis. Table 4 outlines recommended samples, their purposes, and the primary pathogens to target. Proper sample handling is essential to ensure diagnostic accuracy. Samples should be refrigerated (4 °C) if processing is delayed, while those intended for long-term molecular analysis should be frozen. Specimens for bacterial culture should not be frozen and should be collected in sterile containers to minimize contamination. All samples should be clearly labeled with animal identification, collection date, and relevant clinical history, and transported to the laboratory as rapidly as possible under a maintained cold chain [13,80].

During the first 24 h, aborting females should be isolated immediately, animal movement restricted, and aborted fetuses and placental tissues removed and disposed of safely. Because several potential abortifacient pathogens are zoonotic, personnel involved in obstetrical procedures, sample collection, cleaning, and disposal should use appropriate personal protective equipment and avoid unnecessary direct exposure to fetuses, placentas, blood, and uterine or vaginal discharges. Strict biosecurity measures, including protective clothing, appropriate hand hygiene, equipment disinfection, and environmental decontamination, should be implemented to reduce both animal-to-animal transmission and occupational exposure. Suspected cases of important zoonotic or notifiable diseases, such as brucellosis or Rift Valley fever, should be promptly reported to the relevant veterinary and public-health authorities [13,80,81].

During the following two weeks, the herd should be systematically evaluated by examining pregnant females, reviewing nutritional status and feed quality, and identifying potential environmental or management-related stressors. Affected animals should receive appropriate supportive care, including treatment for retained fetal membranes or metritis when indicated, while antimicrobial therapy should be guided by clinical findings and laboratory results. If vector-borne diseases are suspected, vector control measures should be implemented, and biosecurity strengthened through quarantine of newly introduced animals, separation of pregnant females, improved sanitation, and restriction of contact with other livestock species [26,58,65,82].

Long-term prevention should focus on integrated herd health management. Vaccination may contribute to the prevention of abortion associated with specific infectious agents; however, its applicability in dromedary camels is highly pathogen- and country-dependent. Vaccine availability, regulatory approval, and evidence of safety and efficacy vary considerably among countries and pathogens. Therefore, the availability of a vaccine for a particular pathogen in cattle, sheep, goats, or other livestock should not be interpreted as evidence that an equivalent, validated vaccination protocol is available or effective in dromedary camels. Where a vaccine is licensed and supported by appropriate evidence for use in camels, vaccination should be incorporated into herd-level disease prevention programs according to national veterinary regulations. For several potential abortifacient pathogens, camel-specific evidence regarding protective efficacy, optimal vaccination schedules, and duration of immunity remains limited. Consequently, vaccination should be considered as one component of an integrated prevention strategy, together with biosecurity, animal movement control, surveillance, diagnostic investigation of abortion cases, and sound herd management [1,83,84].

The practical measures summarized in Table 5 provide an on-farm framework for interrupting transmission, protecting susceptible pregnant females, supporting timely diagnosis, and monitoring the progression of an abortion storm.

## 10. Zoonotic and One Health Considerations

Abortion storms in dromedary camels have important One Health implications because several pathogens involved in reproductive disease are zoonotic or pose recognized occupational risks. *Brucella* spp., *Coxiella burnetii*, *Chlamydia abortus*, and RVFV warrant particular attention. Brucellosis is a well-documented zoonosis with robust evidence linking camel and human exposure, and veterinarians, farmers, and abattoir workers may be exposed when handling infected animals, aborted fetuses, or placental tissues [30,32]. *C. burnetii*, the causative agent of Q fever, is also zoonotic, with high concentrations occurring in reproductive materials such as placenta and amniotic fluids; inhalation of contaminated aerosols represents a primary route of human exposure [85]. *C. abortus* can infect humans following exposure to infected products of abortion or parturition, with pregnant women being particularly vulnerable to severe reproductive consequences [86]. RVFV is a mosquito-borne zoonotic pathogen; human infection may occur through direct or indirect contact with blood or tissues from infected animals, including during assistance at parturition and disposal of infected fetuses or carcasses [87]. Consequently, abortion investigations should be conducted with appropriate personal protective equipment, hygienic handling and safe disposal of abortion materials, and prompt notification of veterinary and public-health authorities when zoonotic or notifiable diseases are suspected.

## 11. Knowledge Gaps and Future Perspectives

Despite increasing recognition of abortion storms as an important cause of reproductive loss in dromedary camels, major knowledge gaps remain. A standardized, camel-specific epidemiological definition of abortion storms is lacking, and validated thresholds based on the proportion of pregnant females aborting within a defined period have not been established, limiting comparability across studies [25,58]. Prospective, longitudinal herd-level studies that quantify abortion incidence using appropriate denominators and standardized case definitions are scarce. Standardized diagnostic algorithms for abortion investigation, including harmonized sampling protocols, are also lacking, and camel-specific diagnostic assays, particularly serological tests adapted from other species, remain poorly validated [88,89,90]. Systematic fetal and placental pathology, including histopathology, is inadequately applied in field investigations [1,25]. The role of mixed infections, co-infections, and subclinical carriers in triggering and sustaining abortion storms remains poorly understood, and molecular epidemiology and genomic tools have been underused for understanding pathogen transmission, evolution, and sources of introduction. Finally, controlled intervention studies evaluating the effectiveness of vaccination, biosecurity, antimicrobial treatment, and herd-management measures in preventing abortion storms are lacking. Addressing these gaps through coordinated, multidisciplinary research is essential to improve diagnosis, prevention, and control of abortion storms in dromedary camels.

## 12. Conclusions

Abortion storms in dromedary camels should be understood as multifactorial events in which well-established, suspected, and emerging agents may interact with host, environmental, and management factors. Prevention and control should therefore be based on evidence-proportional interpretation rather than assuming equivalent etiological roles for all reported pathogens. Early field investigation, appropriate sampling, laboratory confirmation, and integrated herd health management are essential for effective control. Future progress depends on standardized surveillance systems, camel-specific diagnostic tools, and multidisciplinary research to improve prevention and reduce economic losses.

## Figures and Tables

**Table 1:** Summary of field studies on abortion in dromedary camels: clinical findings, diagnostic approaches, etiological agents, sample types, and associated risk factor.

Year(s)	Location	Animals	Management	Clinical Findings	Sample Type	Diagnostic Method	Main Laboratory Findings	Risk Factors	Strength of Etiological Evidence	Reference	Comment
**2002**	Spain (Canary Islands)	Pregnant (n = 3), post-abortion (n = 5), weak/premature calves (n = 8); 6–12 y	Semi-intensive (inferred)	Abortion (6–8 months), neonatal mortality, hyporexia	Blood	Microscopy	*Trypanosoma evansi*, hemolytic anemia	NR	Moderate	Gutierrez et al., 2005 [8]	Outbreak; monitored herd
**2003**	UAE (Dubai)	80 pregnant (8 abortions)	Semi-nomadic (reported)	Mid–late gestation abortion	Fetal tissues	Isolation	Equine rhinitis A virus (10%)	Contact with horses	High	Wernery et al., 2008 [20]	Viral outbreak
**2013**	Sudan	3413 camels (110 herds)	Extensive (inferred)	Abortion, retained placenta	Serum	Serology + bacteriology	*Brucella* spp. (~8%)	Age > 4 y, abortion history	Moderate	Musa & Shigidi, 2013 [21]	Large pastoral herds
**2013–2016**	KSA	182 camels (103 herds)	Mixed (semi-intensive/extensive)	Abortion storms, uterine disorders	Uterine swabs	PCR	*Coxiella burnetii* (36%), *Campylobacter* spp. (27%), *Brucella* spp. (17%), *Salmonella* spp. (13%), *Chlamydia* spp. (7%)	Abortion history	High	Khalafalla et al., 2017 [13]	Multi-pathogen
**2017**	KSA	245 camels	Semi-intensive (inferred)	History of abortion	Serum	ELISA	*Chlamydia* spp. (19.6%)	NR	Low	Zaher et al., 2018 [10]	Serology only
**2018**	KSA	210 camels	Semi-intensive (inferred)	Late-term abortion	Serum	ELISA + PCR	*Chlamydia abortus* (33.8%)	NR	Moderate	Abdullah and Hameed, 2021 [2]	Outbreak
**2018**	Egypt	315 camels	Semi-intensive (inferred)	History of abortion	Serum	ELISA	*Coxiella burnetii* (22%)	Age, abortion history	Low	Selim & Ali, 2020 [22]	Serology only
**2020**	Egypt	282 camels	Semi-intensive (inferred)	History of abortion	Serum	ELISA	*Neospora caninum* (10.99%)	Age, autumn, dogs	Low	Selim & Abdelhady 2020 [16]	Serology only
**2020**	Iran	244 fetuses	Semi-intensive (inferred)	Abortion outbreak	Abomasal content	PCR	*Trypanosoma evansi* (16.8%)	NR	High	Nazem et al., 2020 [12]	Fetal confirmation
**2020**	UAE	16 female 2 male	Semi-intensive (inferred)	Abortion outbreak	Blood	buffy coat + ELISA	*Trypanosoma evansi*	NR	Moderate	Wernery et al., 2020 [3]	Outbreak; monitored herd
**2021–2022**	Ethiopia	450 camels	Extensive (inferred)	Abortion, retained placenta	Serum	RBPT + ELISA	*Brucella* spp. (2.9%)	Abortion history	Low	Ahad et al., 2024 [23]	Pastoral system
**2022**	Qatar	248 camels (28 farms)	Semi-intensive (reported/inferred)	History of abortion	Serum	Rose Bengal + ELISA	*Brucella* spp. (20.6%)	Age > 4 y	Low	Alhussain et al., 2022 [24]	Farm-based
**2022–2025**	Algeria	75 herds	Extensive (inferred)	Abortion, stillbirth	Fetal tissues	Histopathology	*Toxoplasma gondii* (4.8%)	Cats	Moderate	Djeddou-Benabid et al., 2026 [25]	Tissue lesions
**2024**	Tunisia	470 camels	Mixed (semi-intensive/extensive)	History of abortion	Serum	ELISA	*Chlamydia* spp. (5.75%), *Brucella* spp. (2.12%)	Age, winter, previous abortion	Low	Selmi et al., 2024 [15]	Multi-region
**2024**	Egypt	410 camels	Semi-intensive (inferred)	History of abortion	Serum	ELISA	*Chlamydia* spp. (6.6%)	Age, herd size, mixed species	Low	Selim et al., 2024a [26]	Mixed species
**2024**	Egypt	400 camels	Semi-intensive (inferred)	History of abortion	Serum	ELISA	BVD virus (4.8%)	Age, prior abortion, mixed species	Low	Selim et al., 2024b [27]	Cross-species

**NR:** Not reported; **Evidence level:** Low = serological evidence or exposure data without direct detection in abortion-associated tissues; Moderate = epidemiological association and/or direct pathogen detection without sufficient pathological or epidemiological evidence to establish causation; High = direct detection of the pathogen in fetal, placental, uterine, or reproductive tissues accompanied by compatible pathological findings and/or strong epidemiological evidence supporting etiological involvement. Detection of a pathogen, including in high-evidence studies, should not be interpreted as definitive proof of causation in the presence of possible co-infections or other contributing factors. Studies reporting individual abortions or exposure data should not be interpreted as documenting an abortion storm. Management system was inferred where not explicitly reported in the original studies.

**Table 2:** Major Risk Factors Potentially Associated with Abortion Storms in Dromedary Camels.

Category	Risk Factor	Mechanism/Impact	Evidence Basis *
Animal-related	Age (young/old)	May influence reproductive efficiency and host susceptibility	Camel-specific
	Stage of gestation	May influence susceptibility to pregnancy loss and pathogen-associated reproductive damage	Proposed/Comparative
	Immune status	May influence ability to control infection and maintain pregnancy	Proposed/Comparative
Herd-level	Large herd size	May increase the number of susceptible animals and opportunities for transmission	Comparative/Proposed
	High stocking density	Increased close contact and environmental contamination	Comparative
	Introduction of new animals	May introduce pathogens into susceptible herds	Camel-specific/Comparative
	Mixed-species farming	May facilitate cross-species pathogen transmission	Comparative/Proposed
Environmental	Seasonality	May alter pathogen exposure, animal movement, vector activity, and reproductive management	Camel-specific/Comparative
	Heat stress	May cause physiological and metabolic stress and potentially affect immune and reproductive function	Comparative/Proposed
	Vector abundance	May increase transmission of vector-borne pathogens	Comparative/Proposed
Management	Poor biosecurity	May facilitate introduction and within-herd spread of infectious agents	Comparative/Proposed
	Improper disposal of fetuses and placentas	May increase environmental contamination and exposure to infectious agents	Camel-specific/Comparative
	Nutritional deficiencies	May impair immune and reproductive function	Comparative/Proposed
	Transport and handling stress	May induce physiological stress and potentially affect immune function	Comparative/Proposed

* Evidence basis: Camel-specific = supported by studies conducted directly in dromedary camels; Comparative = supported primarily by evidence from other livestock species and considered potentially relevant to dromedaries; Proposed = biologically plausible or suggested as a potential contributor but with limited direct evidence in dromedary camels. These categories describe the source and applicability of the available evidence and should not be interpreted as definitive proof of causation.

**Table 3:** Diagnostic Tools Comparison.

Method	Advantages	Limitations	Field Applicability
Serology	Easy, low cost	Cannot confirm active infection	High
PCR	Provide high analytical sensitivity and specificity when appropriately validated for the target pathogen, sample type, and laboratory conditions, but that performance depends on assay design, sample quality, timing of collection, presence of inhibitors, and laboratory procedures.	Expensive, requires lab	Moderate
Culture	Provide strong or definitive evidence of viable pathogen presence	Time-consuming, low sensitivity, requires appropriate biosafety containment	Low
Histopathology	Detects tissue lesions	Requires expertise	Low

**Table 4:** Recommended samples, diagnostic purposes, and target pathogens for laboratory investigation of abortion in dromedary camels.

Sample	Collection and Handling	Diagnostic Purpose	Main Targets
Placenta	Collect representative cotyledons and intercotyledonary membranes aseptically. Submit fresh portions in sterile containers for PCR/culture; place separate representative sections in 10% neutral buffered formalin for histopathology.	Gross examination, histopathology, PCR, bacterial/fungal culture	*Brucella* spp., *Chlamydia abortus*, *Coxiella burnetii*, fungal agents
Fetal abomasal contents	Aspirate aseptically with a sterile syringe or collect the entire abomasum. Fresh sample in sterile container; do not freeze if culture is required.	Bacterial culture, PCR	*Brucella* spp., *Salmonella* spp., *Campylobacter* spp.
Fetal lung	Collect representative fresh tissue aseptically in a sterile container for PCR/viral isolation; separate tissue in formalin if histopathology is indicated.	PCR, viral isolation, histopathology	BVDV, ERVA, RVFV, bacterial pathogens
Fetal liver	Fresh tissue in sterile container for culture/PCR; separate tissue in formalin for histopathology when indicated.	Bacterial culture, PCR, histopathology	*Brucella* spp. and other bacterial pathogens
Fetal spleen	Collect fresh tissue aseptically for PCR; separate tissue in formalin if histopathology is required.	PCR, histopathology	*Trypanosoma evansi*, *Toxoplasma gondii*, viral pathogens
Fetal brain	Fresh tissue in sterile container for PCR; representative tissue in formalin for histopathology when indicated.	PCR, histopathology	*Toxoplasma gondii*, RVFV and other relevant pathogens
Vaginal swab/discharge from dam	Collect using a sterile swab before antimicrobial treatment, preferably from the reproductive tract discharge.	PCR, bacterial culture	*Chlamydia abortus* and other bacterial pathogens
Maternal serum	Collect blood into a plain tube; separate serum and refrigerate if testing is delayed.	Serology	*Brucella* spp., *C. burnetii*, *T. gondii*, BVDV, RVFV
Maternal EDTA blood	Collect aseptically into EDTA tube; maintain appropriate cold-chain conditions.	PCR, hematology	*T. evansi* and other blood-borne pathogens
Whole aborted fetus	Submit the entire fetus, when feasible, under refrigerated conditions and avoid freezing when culture is anticipated.	Complete necropsy, gross pathology, targeted sampling	All potential pathogens

**Table 5:** Practical on-farm measures for control of abortion storms in dromedary camels.

Stage	Practical Measure	Main Objective
First abortion detected	Identify and isolate the affected female from pregnant animals	Reduce exposure
Immediately after abortion	Promptly remove and safely dispose of the fetus, placenta, and heavily contaminated materials	Prevent environmental contamination
Abortion site	Restrict access of animals and unnecessary personnel to contaminated areas	Reduce transmission
Feed and water	Protect feed and water from contamination with reproductive materials and discharges	Reduce environmental/oral exposure
Pregnant contacts	Identify exposed pregnant females and increase clinical monitoring	Facilitate early detection
Diagnostic investigation	Submit fetus and placenta, when available, together with appropriate maternal samples	Establish the etiology
Herd management	Temporarily minimize animal movement, herd mixing, and introduction of replacement animals	Limit pathogen introduction and amplification
Equipment and facilities	Clean and disinfect contaminated equipment, maternity areas, and abortion sites	Interrupt indirect transmission
Breeding males	Evaluate breeding males when epidemiologically indicated and perform appropriate diagnostic testing	Investigate possible reproductive transmission
Personnel	Use appropriate PPE and follow zoonotic-disease precautions	Protect human health
Specific interventions	Apply pathogen-specific treatment or vaccination only when supported by diagnostic evidence and appropriate authorization	Target the underlying cause
Follow-up and surveillance	Record pregnancies at risk, abortions, dates, gestational stages, laboratory findings, and relevant management changes	Monitor outbreak progression and control effectiveness

## Data Availability

The raw data supporting the conclusions of this article will be made available by the authors, without undue reservation.
